# A review on abusive content automatic detection: approaches, challenges and opportunities

**DOI:** 10.7717/peerj-cs.1142

**Published:** 2022-11-09

**Authors:** Bedour Alrashidi, Amani Jamal, Imtiaz Khan, Ali Alkhathlan

**Affiliations:** 1 Department of Computer Science, King Abdul Aziz University, Jeddah, Saudi Arabia; 2 Department of Computer Science, University of Hail, Hail, Saudi Arabia; 3 Department of Computer Science, Cardiff Metropolitan University, Cardiff, UK

**Keywords:** Abusive content, Offensive language, Hate speech, Machine learning, NLP

## Abstract

The increasing use of social media has led to the emergence of a new challenge in the form of abusive content. There are many forms of abusive content such as hate speech, cyberbullying, offensive language, and abusive language. This article will present a review of abusive content automatic detection approaches. Specifically, we are focusing on the recent contributions that were using natural language processing (NLP) technologies to detect the abusive content in social media. Accordingly, we adopt PRISMA flow chart for selecting the related papers and filtering process with some of inclusion and exclusion criteria. Therefore, we select 25 papers for meta-analysis and another 87 papers were cited in this article during the span of 2017–2021. In addition, we searched for the available datasets that are related to abusive content categories in three repositories and we highlighted some points related to the obtained results. Moreover, after a comprehensive review this article propose a new taxonomy of abusive content automatic detection by covering five different aspects and tasks. The proposed taxonomy gives insights and a holistic view of the automatic detection process. Finally, this article discusses and highlights the challenges and opportunities for the abusive content automatic detection problem.

## Introduction

In the last decade, the use of social media platforms such as Twitter, Facebook, YouTube, and Instagram have experienced a sharp increase because of many users joining those platforms daily and sharing their updates. Furthermore, the latest statistics show that social media platforms have increased rapidly, and currently, there are more than 1.6 billion social network users worldwide, with more than 64 percent of internet users accessing social media services online (Statista, 2020). Every second, an uncontrolled number of tweets, posts and comments are posted on social media platforms, making it impossible to track or govern the content of such sites. Cyberspace, on the other hand, is not necessarily safe; it can be a source of throwing insults and abusive content towards other people. Therefore, the automatic detection of abusive content on social media needs serious attention, and it is important to highlight what happens. Based on statistics, there is evidence of the occurrence of abusive content in the social media platform (Duggan, 2017). Abusive language can be triggered by provoking events that arise in anger and hate based on race, gender, or religion (Zhang & Wallace, 2015).

Abusive language detection is an unsolved and challenging problem for the natural language processing community (Caselli et al., 2020). Therefore, abusive content should be monitored and regulated among the researcher communities with support from social media platform authorities and government sectors. Nevertheless, it has been countered by harnessing the power of recent advances in computational linguistics. However, social media platforms cannot control and handle all posts for all users, so there is a need to develop and enhance the performance of abusive content automatic detection models.

Recently, with the advancement of NLP technology, many studies have been performed on automatically detecting abusive content and its variants. Several well-known competitions, *e.g.*, SemEval-2020 (Zampieri et al., 2020), GermEval-2018 (Wiegand, Siegel & Ruppenhofer, 2018), OSACT-2020 (Al-Khalifa et al., 2020) and HASOC-2020 (Dowlagar & Mamidi, 2020), have organized numerous events in the hopes of finding a better solution for automated abusive content identification. Additionally, researchers have populated large-scale databases from many sources in this area, which has fueled field studies.

In this regard, it is important to conduct literature review study in abusive content automatic detection problem. Nevertheless, there are some review papers related to abusive content detection but some of them did not presents the entire process of abusive content detection tasks such as Kaur, Singh & Kaushal (2021). Further investigation can be undertaken to explore and track the effects of the recent approaches, techniques, categorization methods and the annotation process. Moreover, the existing work tackling this problem in general especially the categorization task without considering a fine-grained classification method. For this reason, we believe that this article will give an insightful view of the entire tasks in this problem. Specifically, this article will provide a review of the abusive content automatic detection approaches and tasks.

Subsequently, this article proposes a new taxonomy which covers five different aspects and tasks, we explained them extensively in abusive content automatic detection section. The term taxonomy is defined in Pinchner (2022) as “It is a set of chosen terms use to retrieve on-line content—to make the search and browse capabilities of the content, document or records management systems truly functional. ”. Furthermore, taxonomy is known as “a knowledge Organization System (KOS) or a set of elements, often structured and controlled, which can be used for describing (indexing) objects, browsing collections etc.” (Wikipedia, 2022). The proposed taxonomy was designed and created after a comprehensive review of abusive content automatic detection approaches and tasks. The first task represents the data resources, repositories, and programming languages *e.g.*, social media APIs, the repositories names, the major types of the files, and the programming tools and techniques. The second aspect was related to categorization and annotation tasks, which includes the categorization and the annotation process. The third task represents the preprocessing techniques and the feature representation *e.g.*, data cleaning and feature representation types. The fourth task related to the ML, DL models and approaches. Finally, the fifth task represents the evaluation metrices.

The rest of the article is organized as follows: Background section presents an overview of abusive content categories and its related definitions. The survey methodology section describes the research questions with its flow through the rest of the article and the process of related papers selection. Abusive content automatic detection section details the proposed taxonomy with related tasks and aspects. The research challenges and opportunities section present the research gaps and proposes future directions. Finally, the conclusion section presented.

## Background

The automatic detection of abusive content is a challenging task due to disagreements on different abusive content definitions. Moreover, some content might be hateful to some individuals and not to others, based on their concerned definitions. Therefore, one of our main objectives in this study is to explore possible but solid definitions for abusive content and its related categories. Table 1 presents some examples of abusive content tweets from annotated datasets. Generally, abusive content includes many branches and types; therefore, we summarize the most important main categories as follows: hate speech, cyberbullying, and abusive and offensive language. In addition, we will discover some targeted groups, such as religion, racism, gender, and misogyny. Therefore, we will discuss the abusive content categories and targeted groups in the following sections:

**Table 1:** Examples of some tweets that include abusive contents from different annotated datasets.

**Tweet**	**Abusive**	**Offensive**	**Hate speech**	**Cyberbullying**	**Religious**	**Racism**	**Gender** **and Misogyny**
@user dont love muslim immigrants stupid piece sh** thats cant walk streets u idiot (Ousidhoum et al., 2019).	Yes	Yes	–	–	Yes (Origin target)	–	–
@username You are actually disgusting in these sl** pictures. Your parents are probably embarrassed (Salawu, Lumsden & He, 2021).	–	–	–	Yes	–	–	–
@user 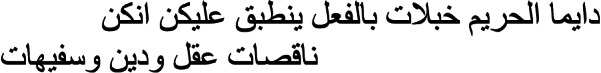 (Duwairi, Hayajneh & Quwaider, 2021). **Translation:** Women are always demented, Indeed you are half brains and crazy.	–	–	Yes	–	–	–	Yes
@user 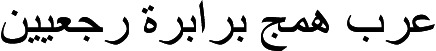 (Duwairi, Hayajneh & Quwaider, 2021). **Translation:** Arabs are brutal, barbaric and retarded.	–	–	Yes	–	–	Yes	–

### Abusive and Offensive language

Abusive language is defined as extremely rude and insulting (Collins, 2017). In addition to the basic definition of abusive language, Fortuna & Nunes (2018) synthesize the earlier definitions by Papegnies et al. (2020), Park & Fung (2017) and Nobata et al. (2016) into the following: “any strongly impolite, rude or hurtful language using profanity, that can show a debasement of someone or something, or show intense emotion”. In addition, Caselli et al. (2020) define abusive language as “hurtful language that a speaker uses to insult or offend another individual or a group of individuals based on their personal qualities, appearance, social status, opinions, statements, or actions”, and they claim that their definition is more comprehensive than the previous definition. However, the term abusive language refers to hurtful language and includes hate speech and offensive language. Many researchers, on the other hand, referred to abusive language as offensive language (Nobata et al., 2016).

### Hate speech

Hate speech is defined by the Cambridge dictionary (CU Press) as “public speech that expresses hate or encourages violence towards a person or group based on something such as race, religion, sex, or sexual orientation”. From the perspective of the research communities in this area, there is no standard and precise definition of hate speech. Therefore, there are some contributions to defining hate speech terminology. For instance, according to Fortuna & Nunes (2018), hate speech is ”the content that promotes violence against individuals or groups based on race or ethnic origin, religion, disability, gender, age, veteran status, and sexual orientation/gender identity”.

### Cyberbullying

Cyberbullying (Dadvar et al., 2015) is the use of digital media to harass an individual or group of individuals, for example, by personal exposure to confidential or false information. It could be considered a criminal offense (Mercado, Chuctaya & Gutierrez, 2018). Furthermore, cyberbullying is defined as the infliction of recurring and repetitive harm using digital media, especially in the world of social networking platforms, allowing an individual the power to embarrass or harm a victim in front of an entire online community  (Mercado, Chuctaya & Gutierrez, 2018). This is widely acknowledged as a severe social problem, particularly among youths (Kowalski et al., 2014).

### Targeted Groups

Target groups are the groups that are targeted or referred to based on the characteristic that includes the members of the community concerned. This trait may be represented in nationality, religion, race, and gender. Therefore, we will discuss the most popular targeted groups as the following:

• Gender and Misogyny

This group comprises any hatred towards a specific gender or devaluation depending on a person’s gender. Any post that offends a specific gender falls into this group. It also encompasses all forms of misogyny. Furthermore, misogynistic speech is a type of abusive language that may be summarized as hate speech directed towards women, and it has become a growing concern in recent years (Pamungkas, Basile & Patti, 2020).

• Religious

This group includes any kind of religious discrimination, such as Islamic sects, calling for atheism, anti-Christian and their respective denominations or anti-Hinduism and other religions. An example is upsetting someone because he or she is a member of a particular tribe, area, or country. Additionally, Albadi, Kurdi & Mishra (2018) mentioned that religious hate speech is considered a motive for crimes in countries with the highest number of social crimes.

• Racism

This group is related to any form of racial offense or tribalism, regionalism, xenophobia, particularly among migrant workers, and nativism hostility towards immigrants and refugees, and any prejudice against a particular tribe or territory falls under this group. An example is upsetting someone because he or she belongs to a certain tribe, area, or country, or it can manifest as bias towards a certain tribe (Al-Hassan & Al-Dossari, 2021).

## Survey Methodology

This article aims to investigate three main research questions and develop a holistic view of abusive content detection problem by proposing a taxonomy that highlights its related aspects and tasks. Specifically, we are adopting Exploratory survey type which can be used to become more familiar with a topic, to explore it, and to try out preliminary concepts about it (Pinsonneault & Kraemer, 1993).

In this regard, we are addressing the following questions:

**First,** what are the strategies and approaches used to detect and categorize abusive contents?

**Second,** whether the approaches adopted for annotation procedure on available open-source datasets are viable?

**Third,** what are the challenges and limitations still exist in the proposed automatic detection approaches and solutions of abusive contents?

Subsequently, we discussed and organized these questions in different sections as follows:

Abusive content automatic detection section presents the answer of the first and second questions. In brief, we investigate and discuss the abusive content categories and its automatic detection approaches and tasks with the evaluation metrics. Conceptually, this section details the proposed taxonomy with its related tasks and other aspects. The third question was investigated and extensively discussed in research challenges and opportunities section.

**Figure 1:**
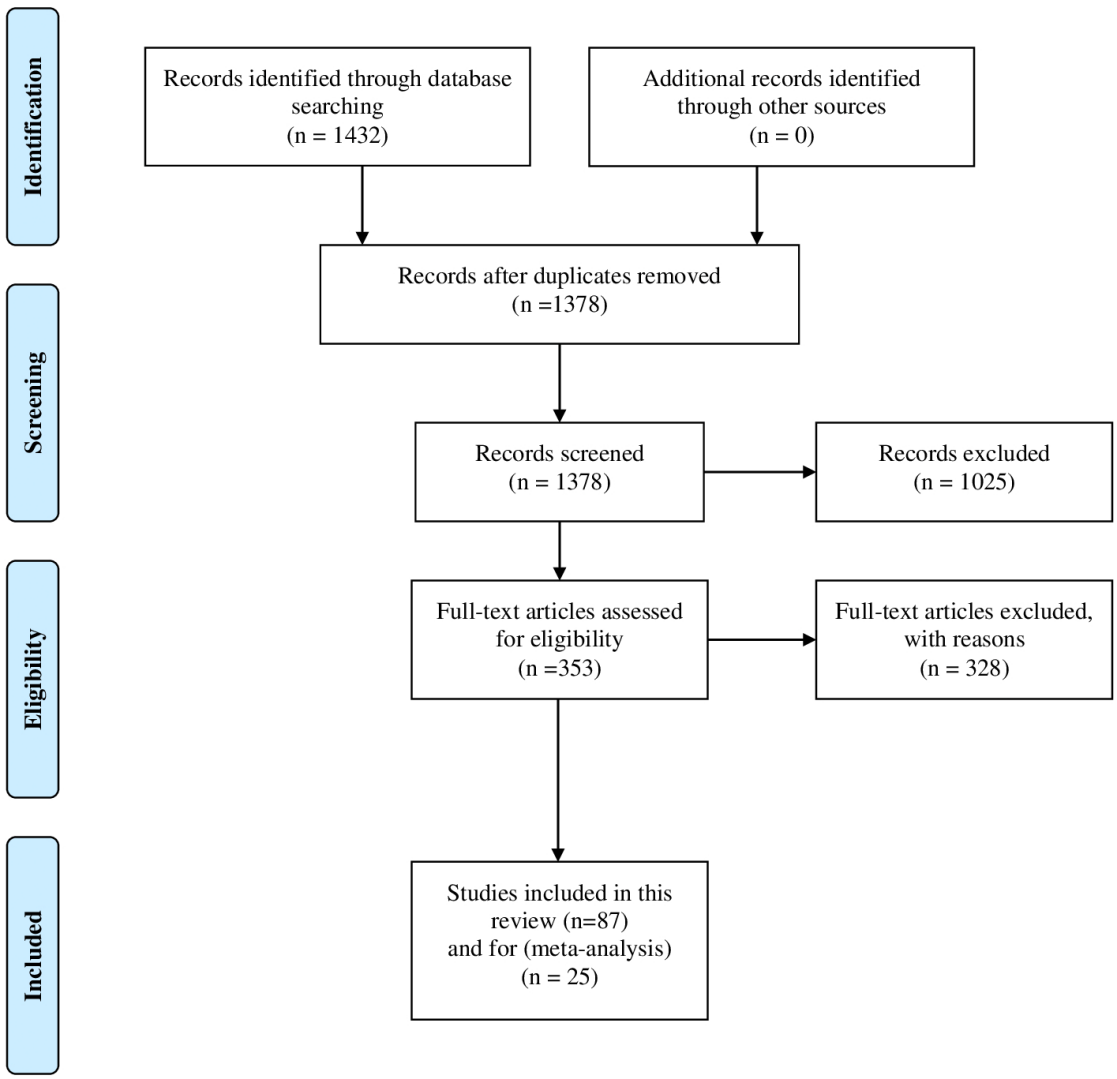
The PRISMA flowchart which illustrates the inclusion and exclusion process.

Mainly, all the discussed works in this study were collected from different academic search engines such as IEEE Explorer (https://ieeexplore.ieee.org/Xplore/home.jsp), ACM (https://www.acm.org/), ACL (https://www.aclweb.org/portal/), arXiv (https://arxiv.org), and Google Scholar (https://scholar.google.com/). Besides the academic search engines, some of journal articles were also discussed and reviewed in this study. To narrow down the scope of our research the following keywords were used to collect the relative articles of main types of abusive content namely: “Abusive language”, “Offensive Language”, “Hate Speech”, and “Cyberbullying”, we used “AND” and “OR” Boolean operators in order to combine the relative articles. Furthermore, we also focused on the recent contributions in this area and for this purpose we used PRISMA (Moher et al.) flow chart to highlight the number of records identified through database searching and the filtering process as it summarized in Fig. 1.

Subsequently, we used some inclusion and exclusion criteria as the following points:

• Inclusion criteria:

- Papers published within the period from January 2017 to December 2021.

- Papers that are related to abusive content detection and its antisocial behavior topics which also contains theoretical and empirical analysis.

• Exclusion criteria:

- Papers that are related to abusive content detection but not in the field of computer science, especially in NLP *e.g.*, (Cyberbullying in high school, Cyberbullying from psychological and legal perspectives...etc.).

- Papers that are related to abusive content detection but not contains technical contents or with no clear analysis.

Initially we obtained 1,432 papers from the academic search engines that mentioned before. Since we have collected the data from different resources, we removed the duplicate records and proceeded the remaining papers 1,378 to the filtering process. Then, we exclude 1,025 paper that were matched the exclusion criteria. The remaining articles 353 were considered for full text review. Finally, 25 papers were selected for meta-analysis and another 87 papers were cited throughout the rest of the paper. The selected articles were with clear objectives, methodologies, analysis, and solid results.

### Abusive content automatic detection

**Figure 2:**
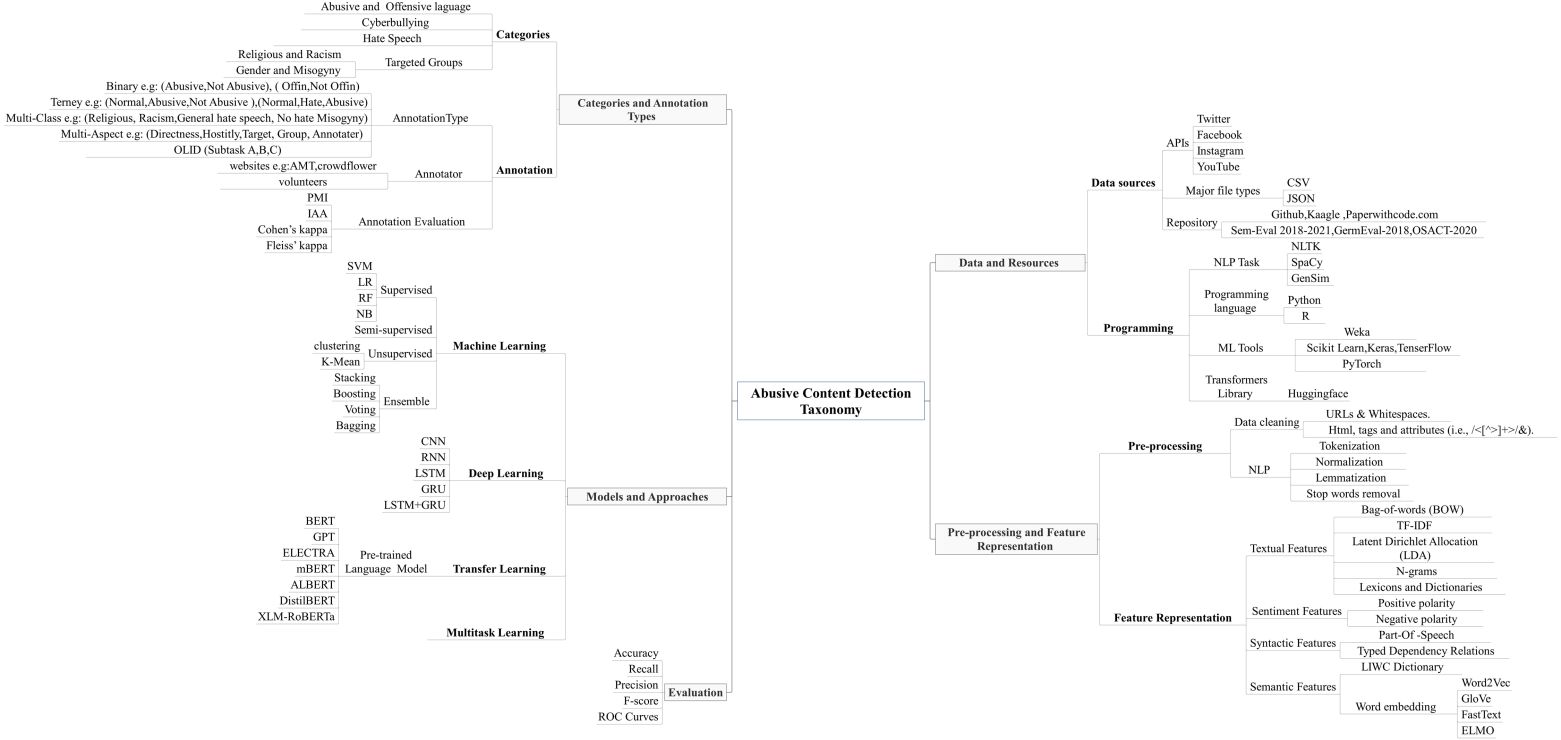
The proposed taxonomy of abusive content detection which illustrates the five main aspects and tasks.

Recently, there has been a noticeable increase in the research studies of abusive content detection that have been conducted by using different resources and approaches. However, to investigate and explore the abusive content detection problem, our main aim in this study is to propose a taxonomy in Fig. 2 that illustrates five different aspects and tasks. Therefore, the proposed taxonomy was designed after an extensive and comprehensive review of previous abusive content detection studies and their related categories from all discussed papers in this study. In addition to the discussed papers and to understand the cutting edge in this area to track the latest approaches, resources, statistics, techniques, and methods, we will also consider recent survey papers. Specifically, narrative review papers (Al-Hassan & Al-Dossari, 2019; Mishra, Yannakoudakis & Shutova, 2019; Schmidt & Wiegand, 2017), systematic review papers (Fortuna & Nunes, 2018; Poletto et al., 2021) and more recent systematic review articles (Jahan & Oussalah, 2021). In this section, we will discuss the proposed taxonomy, which covers five different aspects and tasks:

### Data and resources

The collected datasets for abusive content detection tasks originated from various social media platforms and were stored in different repositories. Popular social media platforms such as Twitter, Facebook, YouTube, and Instagram were the main sources used to collect the data due to the nature of those platforms, as they open the door for hate speech, cyberbullying, abusive and offensive language. Furthermore, previous studies and shared task completions, such as SemEval, GermEval and OSACT, collected the data from the main sources by using social media APIs. Then, after several steps for data preparation and annotation, they used to store and share the collected datasets by using repositories such as GitHub (https://github.com/), Kaggle (https://www.kaggle.com/) and Paperswithcode.com (https://paperswithcode.com/). In this part, we will discuss the second question for this study, which aims to explore the available open-source datasets and the annotation procedure task.

We used four keywords to check the availability of the datasets in GitHub, Paperswithcode.com and Kaggle repositories without considering specific language. Specifically, we searched for datasets that are publicly available. The keywords used were “Abusive language”, “Offensive Language”, “Hate Speech” and “Cyberbullying”. The results in Table 2 illustrate that there are more than 2.5k datasets available in the repositories; hence, it is difficult to analyze all the datasets in the repositories. However, we note some points during the research as follows:

**Table 2:** The available datasets of abusive content types from different repositories.

**Keywords**	**Repository Name**		
	**GitHub**	**Kaggle**	**Paperswithcode.com**
Abusive Language	122^a^	14^b^	7^c^
Offensive Language	312^d^	32^e^	91^f^
Hate Speech	1856^g^	60^h^	79^i^
Cyberbullying	568^j^	10^k^	58^l^

**Notes.**

a “Search Abusive Language GitHub.” [Online]. Available: https://github.com/search?q=Abusive+Language.

b “Abusive Language _ Kaggle.” [Online]. Available: https://www.kaggle.com/search?q=abusive+language+in%3Adatasets.

c “Abusive Language _ Papers With Code.” [Online]. Available: https://paperswithcode.com/task/abusive-language.

d “Search Offensive Language GitHub.” [Online]. Available: https://github.com/search?q=Offensive+Language.

e “Offensive Language_Kaggle.” [Online]. Available: https://www.kaggle.com/search?q=Offensive+Language.

f “Offensive Language _ Papers With Code.” [Online]. Available: https://paperswithcode.com/search?q_meta=&q_type=&q=Offensive+Language.

g “Search hate speech GitHub.” [Online]. Available: https://github.com/search?q=Hate+Speech.

h “Hate Speech_kaggle.” [Online]. Available: https://www.kaggle.com/search?q=Hate+Speech.

i “Hate Speech Dataset _ Papers With Code.” [Online]. Available: https://paperswithcode.com/task/hate-speech-detection.

j “Search Cyberbullying GitHub.” [Online]. Available: https://github.com/search?q=Cyberbullying.

k “Cyberbullying_ Kaggle.” [Online]. Available: https://www.kaggle.com/search?q=Cyberbullying+in%3Adatasets.

l “Search for Cyberbullying _ Papers With Code. https://paperswithcode.com/search?q_meta=q_type=q=Cyberbullying.

First, the varieties of subtopics in the same resource. For example, some datasets used sentiment analysis to detect hate speech. Second, many of the available datasets used binary and ternary classification  (Alakrot, Murray & Nikolov, 2018a; De Gibert et al., 2019; Mubarak & Darwish, 2017). Third, it is observed that the dataset sizes are small and did not exceed approximately 100k tweets or comments (Mubarak & Darwish, 2017; Basile et al., 2019). Fourth, a study collected resources and benchmark corpora for hate speech detection (Poletto et al., 2021), and they concluded that biases in corpora design and annotation are a significant issue.

On the other hand, there are some studies that investigate the available dataset for this area. For example, Jahan & Oussalah (2021) investigated 69 hate speech datasets and found that the existing efforts provided a variety of challenges in terms of dataset preparation. Generally, researchers begin by gathering and annotating new comments from social media or by referring to older datasets. Due to the possibility of tweet removal, obtaining an old dataset from Twitter is not always viable. This slows down the research since there are fewer data available, making it more difficult to compare the results of different investigations. Furthermore, 55% of the datasets provided are limited in size and contain only a small amount of hate content.

Another systematic review study in Poletto et al. (2021) they concentrated on hate speech detection resources and benchmark corpora. Their survey reveals that multiple interrelated processes are at stake. The field would highly benefit from a shared, data-driven taxonomy that highlights how all these concepts are linked and how they differ from one another. This would provide a common framework for researchers who want to investigate either the phenomenon at large or one of its many facets.

In addition to automatic detection models and approaches, many tools and programming techniques have been used to perform several tasks. For example, NLP tasks were performed using the Natural Language Toolkit (NLTK) (Rizos, Hemker & Schuller, 2019; Huang, Singh & Atrey, 2014; Pawar et al., 2018), SpaCy (Salminen et al., 2020; Ribeiro et al., 2018) and GenSim (Rizos, Hemker & Schuller, 2019; Chowdhury et al., 2019; Kamble & Joshi, 2018). Furthermore, the majority of the work has been performed using the Python programming language, and some of works done by using R language also some studies have also used the Weka tool to train their models and achieve the results (Huang, Singh & Atrey, 2014; Pericherla & Ilavarasan, 2021; Rachid, Azza & Ben Ghezala, 2020). Recently, researchers used Huggingface.co to import transformer models to perform pretraining and fine-tuning strategies. Transformer models are included in a Python-based library that has an API for using many well-known transformer architectures, such as BERT, which obtain state-of-the-art results on a variety of NLP tasks.

### Categories and annotation types

Data annotation is the process of data labelling of different abusive content categories; it refers to what types or strategies have been followed to annotate the dataset. We summarize the annotation schema in Table 3, which illustrates the different types of annotations with the description for each type. Furthermore, most of the studies in the abusive content field used binary and ternary types. However, multiclass types were used in limited studies in Duwairi, Hayajneh & Quwaider (2021), Al-Hassan & Al-Dossari (2021), ElSherief et al. (2018), and only one study used the multi aspect type in a multilingual study (Ousidhoum et al., 2019). Moreover, a hierarchical multi annotation task called the Offensive Language Identification Dataset (OLID) schema (Zampieri et al., 2020) was used in different studies, such as Zampieri et al. (2020) and Wiedemann, Yimam & Biemann (2020).

### Availability of standard guideline for the annotator

There are various ways to annotate the datasets, and some of the researchers used the Hatebase website (https://hatebase.org) to extract and collect hate terms from their database for annotation purposes. Furthermore, some of the researchers have used the popular CrowdFlower (https://visit.figure-eight.com/People-Powered-Data-Enrichment_T) site for tweet online annotation (Burnap & Williams, 2016 and Davidson et al., 2017), which offers a paid online service where annotators and tweets may be selected, and annotator findings can be approved or rejected. Other researchers used Amazon Mechanical Turk (AMT) (https://www.mturk.com/) to annotate their dataset (Ousidhoum et al., 2019). Manual annotation is another option, but it requires unbiased annotators who volunteer to annotate the tweets. Annotators can be researchers themselves, such as Waseem & Hovy (2016) and Magu, Joshi & Luo (2017), as well as volunteers  (Gitari et al., 2015). Moreover, some researchers designed annotation guidelines to ensure that all annotators had the same perspectives (Alshalan & Al-Khalifa, 2020; Mulki et al., 2019).

**Table 3:** The annotation schema that was used to annotate different abusive content datasets.

**Annotation Type**	**Description**
Binary	Classify the Text into two labels, *e.g.*, Abusive, Not Abusive.
Ternary	Classify the Text into three labels, *e.g.*, Abusive, Not Abusive, Normal.
Multi class	Classify the Text into multiple labels, *e.g.*, Abusive, Racism, Misogyny, Religious Discrimination, Normal.
Multi aspect	Classify the Text into multiple aspects, *e.g.*,
	Directness → Direct, Indirect.
	Hostility → Hated, Abusive, Offensive, Disrespectful, Fearful, Normal.
	Target → Origin, Gender, Sexual Orientation, Religion, Disability, Other.
	Group → Individual, Other, Women, Special needs, African descent.
	Annotator →Disgust, Shock, Anger, Sadness, Fear, Confusion, Indifference.
OLID	Classify the Text into three subtasks:
	Subtask A: Offensive Language Identification →Is the text offensive (OFF) or not offensive (NOT).
	Subtask B: Automatic categorization of offense types → Is the offensive text targeted (TIN) or untargeted (UNT).
	Subtask C: Offense target identification →Who or what is the target of the offensive content, Individual target (IND) or Group target (GRP).

### Annotation evaluation

Abusive content detection is challenging and often subject to human prejudices and ambiguities between different categories. Therefore, the annotation procedures need to be evaluated. In particular, there are some measurements and agreements designed for determining the inter annotator reliability of human judgements on affective text, such as the following:

• Pointwise Mutual Information (PMI)

To evaluate how distinctive the vocabulary of the collected dataset is with respect to each class category, the study in Mulki et al. (2019) conducted word-class correlation calculations, and they calculated the PMI for each word towards its relevant category such that for a word w and a class c, PMI is calculated as in Eq. (1):

(1) \begin{eqnarray*}PMIc(w)=\text{log}( \frac{Pc(w)}{Pc} )\end{eqnarray*}

where:

• *w* is a word.
• c is a class.
• Pc(*w*) represents the appearance of word *w* in the tweets of class c.
• Pc refers to the number of tweets of class c.

• Inter annotator Agreement (IAA)

IAA measures have the capability of estimating the reliability of annotations to some extent on the allocated categories. The approach selected to measure the agreement determines the extent. According to  Artstein & Poesio (2008), they suggest that the weighted coefficients can be significant in certain cases of disagreements. The approach selected to measure the agreement determines the extent. A commonly used agreement coefficient in annotation reliability is Krippendorff’s *α*, which is founded on the assumption that by examining the overall judgement distributions despite the respective annotator that produced the judgements, the expected agreement can be calculated.

Using Krippendorff’s *α* value, the annotation can always be deduced as follows:

Good: for the data annotations with agreement values between 0.8 and 1.

Tentative: for the data annotation with agreement values between 0.67 and 0.8.

Discarded: for the data annotation with an agreement value below 0.67.

For instance, the study by Mulki et al. (2019) using the L-HSAB dataset found a Krippendorff’s *α* value of 76.5%, showing that there was agreement with the minority group, with no consideration of the majority group.

• Cohen’s kappa

The chance agreement can also be determined by Cohen’s kappa  (Artstein & Poesio, 2008) metric. Cohen’s kappa coefficient (*κ*) is a statistical metric Eq. (2) used in measuring the reliability between annotators in qualitative cases. It is characterized by robustness compared to other measures that simply calculate the percent agreements. This metric considers the possibility of the agreement taking place by chance. It operates as a pairwise reliability metric between two annotators. Different studies, such as De Gibert et al. (2019), Mulki et al. (2019), Chatzakou et al. (2017), have utilized this metric to assess annotation tasks.

(2) \begin{eqnarray*}\text{kappa coefficient}(\kappa )= \frac{Po-Pe}{1-Pe} \end{eqnarray*}

where:

- Po is the relative observed agreement among annotators (identical to the accuracy).

- Pe is the hypothetical probability of chance agreement.

• Fleiss’ kappa

The agreement reliability between a fixed number of annotators can be evaluated using Fleiss’ kappa, especially when assigning categorical ratings to many items. Fleiss’ kappa can be described as a simplification of Scott’s pi (*π*) assessment measure for two raters expounded for numerous raters. Unlike Cohen’s kappa and Scott’s pi, which apply for only two annotators, Fleiss’s kappa can apply for any number of annotators and provides categorical ratings to a static number of items. The measure is applied in different studies, such as  Caselli et al. (2020), Duwairi, Hayajneh & Quwaider (2021), and Mubarak et al. (2021).

### Preprocessing and representation

Preprocessing is ais a crucial stage in the data cleaning. Abusive content in social media is considered unstructured text, so it must first transform into a format that allows the classification algorithms to complete the task.

The most common processes in NLP and used in abusive content and hate speech detection are tokenization, normalization, lemmatization and stop word removal in different studies (Alshalan & Al-Khalifa, 2020; Al-Khalifa, Aljarah & Abushariah, 2020). In the reviewed literature, most of the works used the NLTK library to tokenize, remove stop words, remove unwanted characters, correct misspelling lemmatizations and/or stem the raw data. Additionally, more steps were typically applied, such as replacing user mentions, URLs, and hashtags with special characters, as well as removing duplicates. However, recent pretrained models, such as BERT, require a change in the preprocessing steps, as stemming is no longer needed.

Selecting the right features to solve the abusive content detection problem is one of the most challenging tasks, and the features include textual, syntactic, sentiment and semantic representation methods. To employ classification algorithms in automatic detection tasks, the general features of the corpus need to be specified. There are many types of feature representations, and we will explain the most important ones that have been used in abusive content detection problems as the following:

• Dictionaries and Lexicons

This feature is most commonly used in unsupervised machine learning (Assiri, Emam & Al-Dossari, 2018). By utilizing corpora and lexical resources, the detection of profane phrases was addressed by Wiegand et al. (2018); they built their lexicon using a variety of features and a general-purpose lexical resource. Using a shared profanity list from the website phorum.org , Sood, Antin & Churchill (2012) assessed the efficacy of a lexicon-based strategy; they created a system that flags a comment as offensive if it contains any of the words on the phorum.org list and found that misspellings, inability to adapt to evolving offensive language, and the context-specific nature of profanity are the three main reasons for the technique’s poor performance.

• Textual Features

Text features capture the patterns that exist in the text, which the machine learning models can then use to learn from the data. Various types of text features have been proposed in the literature such as bag of words (BOW), term frequency-inverse document frequency (TF-IDF), and n-grams. Some types of text features were used in the same study to compare better performance. For instance, Chen et al. (2012) and Nobata et al. (2016) have proven that n-grams outperform BOW characteristics. There are also different content-based aspects that have been employed in previous studies, including comment length (Dadvar et al., 2015; Davidson et al., 2017), ratio of capital letters (Huang, Singh & Atrey, 2014; Dadvar, Trieschnigg & De Jong, 2014), the use of special characters (Chatzakou et al., 2017), and number of emoticons (Dadvar et al., 2015). Another textual feature called latent Dirichlet allocation (LDA) is a type of topic modelling approach that uses probability. It functions by estimating the latent topics in a set of data, thus enabling the use of these latent topics as features; in part of the words, it was used in offensive language detection over a large-scale Twitter corpus (Xiang et al., 2012).

• Semantic Features

The semantic features are theoretical units of meaning-holding components used to express the meaning of words; these characteristics are extremely important in establishing the type of lexical relationship that occurs between words in a language. LIWC (linguistic inquiry and word count) classes are utilized by researchers to detect abusive content since they provide generalizations of patterns based on semantic information (Al-Garadi, Varathan & Ravana, 2016; Cheng, Danescu-Niculescu-Mizil & Leskovec, 2015). In addition, word embeddings, which allow words with similar meanings to be represented similarly, have lately been used in a number of studies (Djuric et al., 2015; Zhao, Zhou & Mao, 2016). Commonly used word embedding methods include FastText, Word2Vec, and GloVe. The three types represent words by using vectors in a way that captures meaning-related and semantic associations and grammar-based or syntactic correlations. However, this limits the methods, as they cannot capture polysemy correlations. This indicates that for the same word, with varied meanings based on dissimilar contexts, the corresponding represented vectors remain constant. Another word embedding model called embedding from language models (ELMO) has several merits. According to Zhou et al. (2020), ELMO embedding has a better performance compared to CNN when applied. Nevertheless, ELMO comparison with other methods is still inconclusive and limited because it is a novel technology. On the other hand, in comparison to word-level deep networks, character-level text processing may concentrate less emphasis on recording high-level associations between words, and this approach is significantly more compact and uses fewer memory resources (Wullach, Adler & Minkov, 2021; Zhang, Robinson & Tepper, 2018). There are some character-level approaches, such as Canine (Clark et al., 2021), CharBert (Ma et al., 2020), CharacterBERT (El Boukkouri et al., 2021), and Charformer models (Tay et al., 2022), but those approaches are rarely used for abusive content detection tasks.

• Syntactic Features

Part-of-speech (POS) tagging, and dependency relations are two syntactic characteristics that are commonly employed. These characteristics capture the sort of words a user used in a certain comment (Xu & Zhu, 2010). A heavy usage of adjectives, for example, should be suggestive of conveying a viewpoint. Many researchers view the use of first- and second-person pronouns in postings as a feature since they give information about who the material is intended for. A comment using an unpleasant term plus a second-person pronoun such as “you” or “yourself” is very certainly intended to irritate other users  (Nobata et al., 2016; Dadvar et al., 2015; Chen et al., 2012; Al-Garadi, Varathan & Ravana, 2016).

• Sentiment Features

The research community has also investigated sentiment features for identifying abusive language since it might be to led to social psychological phenomena like aggressive and antisocial behavior. For example , in the study Chatzakou et al. (2017) they employed the SentiStrength tool to determine the sentiment of the text , as this tool are used to detect the positive and negative sentiment. Another study, in their feature set for classification, Yin et al. (2009) included the presence of pronouns and foul language as sentiment features. Justo et al. (2014) used SenticNet−3.0 (Cambria & Olsher, 2014) to identify each post’s positive and negative polarity. Recently, there are state-of-the-art studies applied some sentiment analysis features. For instance, in the study Asif et al. (2020) they focused on the sentimental analysis of social media multilingual textual data to discover the intensity of the sentiments of extremism. More recent ,in the study Ali et al. (2022) they investigated the correlation between how news stories covered by mainstream news channels impede the hate speech/Islamophobic sentiment.

### Models and approaches

AI methods and techniques, including ML, DL and recently pretrained language models, were an essential step to detect abusive content. This section will provide a comparative and quantitative analysis among different ML, DL, TL automated detection models. Therefore, we analyzed 25 articles in Table 4 from the previous contributions in different languages. The collected articles strategy was mentioned in survey methodology section. Generally, with the growth of DL and TL technologies, there has been a significant shift in abusive content analysis methodologies. However, we will also discuss those models briefly in the following:

**Table 4:** Summary of the selected 25 papers on abusive content detection in different languages and with illustrations of Platform, Category, Feature representation, Algorithms, and Performance measurements.

**Paper/Year**	**Language**	**Platform**	**Category**	**Features Representation**	**Algorithm**	**Performance Measurement**
Park & Fung (2017)	English	Twitter	Abusive	Character and Word2vec	Hybrid CNN	**-Precision:** 0.71 **-Recall:** 0.75 **-F1-Score**: 0.73
Chen, McKeever & Delany (2017)	English	YouTube, Myspace, Slashdot	Abusive	Word embeddings	FastText	**-Recall:**0.76
Abozinadah & Jones (2017)	Arabic	Twitter	Abusive	PageRank (PR) algorithm, Semantic Orientation (SO) algorithm	SVM	**-Accuracy:** 96
Badjatiya et al. (2017)	English	Twitter	Sexist, Racist	Fast Text, GloVe Random Embedding-IDF, BOW	LR, SVM, CNN, LSTM and GBDT	**-Precision:** 0.93 **-Recall:** 0.93 **-F1-Score: 0.93**
Haidar, Chamoun & Serhrouchni (2017)	Arabic	Facebook, Twitter	Cyberbullying (Yes, No)	Tweet to SentiStrength, Feature Vector	SVM	**-Precision: 0**.93 **-Recall:** 0.94 **-F1-Score:** 0.92
Özel et al. (2017)	Turkish	Twitter, Instagram	Hate	BOW	Naïve Bayes	**-F1-Score:** 0.79
Alfina et al. (2018)	Indonesian	Twitter	Hate, Non-hate	BOW and n-gram	Random Forest	**-F1-Score:** 0.93
Wiegand, Siegel & Ruppenhofer (2018)	English	Twitter, Wikipedia, UseNet	Abusive	Lexical, linguistics and word embedding	SVM	**-Precision:** 0.82 **-Recall:** 0.80 **-F1-Score:** 0.81
Watanabe, Bouazizi & Ohtsuki (2018)	English	Twitter	Hate, Offensive	Sentiment-Based, Semantic, Unigram	J48graft	**-Precision:** 0.79 **-Recall:** 0.78 **-F1-Score:** 0.78
Pawar et al. (2018)	English	Formspring	Cyberbullying	BOW	Stochastic Gradient Descent	**-F1-Score:** .90
Malmasi & Zampieri (2018)	English	Twitter	Hate, offensive	N-grams, Skip-grams, hierarchical, word clusters	SVM	**-Precision:** 0.78 **-Recall:** 0.80 **-F1-Score:** 0.79
Pitsilis, Ramampiaro & Langseth (2018)	English	Twitter	Racism or Sexism	Word-based frequency, vectorization	RNN and LSTM	**-Precision:** 0.90 **-Recall:** 0.87 **-F1-Score:** 0.88
Fernandez & Alani (2018)	English	Twitter	Radicalization	Semantic Context	SVM	**-Precision:** 0.85 **-Recall:** 0.84 **-F1-Score:** 0.85
Alhuzali & Abdul-Mageed (2018)	Arabic	Twitter	Adult, Regular user	Lexicon, N-grams, bag-of- means (BOM)	SVM	**-Accuracy:** 79 **-Precision:** 0.70 -**Recall:** 0.93 **-F1-Score:** 0.78
Alakrot, Murray & Nikolov (2018b)	Arabic	YouTube	Offensive, Inoffensive	N-gram	SVM	**- Accuracy:** 90.05
Kamble & Joshi (2018)	Code-mixed English and Hindi	Twitter	Hate speech	Word2Vec	LSTM, BiLSTM, CNN	**-Precision:** 0.83 **-Recall:** 0.78 **-F1-Score:** 0.80
Albadi, Kurdi & Mishra (2018)	Arabic	Twitter	Religious hate, Not hate	Word embeddings (AraVec)	GRU-based RNN	**-AUROC:** 0.84
Rizos, Hemker & Schuller (2019)	English	Twitter	Hate speech	FastText, Word2Vec, GloVe	CNN, LSTM, GRU	**-F1-Score**: 0.69
Ousidhoum et al. (2019)	English	Twitter	Sexual orientation, Religion, Disability	BOW	LR, BiLSTM	**-F1-Score**: 0.94
Zhang & Luo (2019)	English	Twitter	Racism, Sexism	Word embeddings	CNN+GRU	**-F1-Score**: 0.94
Jaki & De Smedt (2019)	German	Twitter	Radicalization	Skip-grams and Character tri-grams	K-means, single-layer averaged Perceptron	**-Precision:** 0.84 **-Recall:** 0.83 **-F1-Score:** 0.84
Alshalan & Al-Khalifa (2020)	Arabic	Twitter	–	–	CNN, GRU, CNN+GRU, BERT	**-F1-Score:** 0.79 **-AUROC:** 0.89
Alatawi, Alhothali & Moria (2021)	English	Twitter	Hate, not Hate	Word2Vec	BiLSTM-BERT	**-F1-Score**: 0.80
Al-Hassan & Al-Dossari (2021)	Arabic	Twitter	Hate, Racism, Sexism	Keras word embedding	LSTM, GURU, CNN+GRU, CNN+LSTM	**-Precision:** 0.72 **-Recall:** 0.75 **-F1-Score:** 0.73
Duwairi, Hayajneh & Quwaider (2021)	Arabic	Twitter	Hate, Abusive, Misogyny, Racism, Religious Discrimination	CNN, CBOW	CNN, CNN-LSTM, and BiLSTM-CNN	**-Accuracy:** 74

• Machine Learning (ML)

Much of the existing work on abusive content detection, however, focuses on using supervised machine learning (Alakrot, Murray & Nikolov, 2018a; Haidar, Chamoun & Serhrouchni, 2017; Gaydhani et al., 2020 and Kanan, Aldaaja & Hawashin, 2020). Furthermore, in a semi supervised study (Xiang et al., 2012), they argued that their approach can be a good alternative to costly supervised approaches for detecting hate speech since it substitutes costly manual annotation with an automatically generated feature. For the unsupervised approach,  Gitari et al. (2015) developed their lexicon and used a bootstrapping strategy, starting with a small seed of hatred verbs and progressively expanding it, and the best outcomes were obtained when they included semantic hate features. In study Di Capua, Di Nardo & Petrosino (2016), they suggested an unsupervised technique based on self-organizing maps (SOMs) that can cluster documents including bully traces efficiently. For an ensemble approach such as stacking, boosting, voting and bagging, many studies apply those approaches to improve the classification result (Haralabopoulos, Anagnostopoulos & McAuley, 2020; Raisi & Huang, 2018).

• Deep Learning (DL)

Deep learning is a branch of machine learning based on a complex artificial neural network. There are many types of DL neural networks, such as conventional neural networks (CNNs), recurrent neural networks (RNNs), gated recurrent units (GRUs) and bidirectional long short-term memory (Bi-LSTM). To combat the problem of abusive content and hate speech identification, researchers have turned to DL algorithms. Recently, many of the studies used various DL approaches as they have gained significant popularity in the research community, and they achieved outperformance (Duwairi, Hayajneh & Quwaider, 2021; Alshalan & Al-Khalifa, 2020; Mohaouchane, Mourhir & Nikolov, 2019). Several studies have demonstrated the superiority of DL models, such as CNNs employing word2Vec, GloVe, FastText, and other embeddings, which outperform standard machine learning models, such as SVM, LR, NB, and RF models (Dowlagar & Mamidi, 2020; Badjatiya et al., 2017). Furthermore, recent studies have found that combining two or more deep learning models outperforms using a single deep learning model. For instance, CNN+LSTM and CNN+GRU outperformed the single application of LSTM and CNN (Al-Hassan & Al-Dossari, 2021).

• Transfer Learning (TL)

Transfer learning is a notion in the machine learning area in which prior knowledge learned from one domain and task is applied to solve a problem from a different domain and task that is connected in some way. The first attempts to apply the transfer learning approach to adjust to the best performance in NLP tasks were word embedding models, which encode and represent an entity such as a word, sentence, and document to a fixed-length vector. Recently, TL approaches were applied in some studies for abusive content detection, such as Mozafari, Farahbakhsh & Crespi (2020). In addition, different contextual based pretrained and transformer models were released by Google AI and other companies and achieved state-of-the-art performance in many NLP tasks. This section is entirely dedicated to the very important topics of the transformer and especially the BERT models.

***The transformer*** is a neural network architecture used in sequence modelling that was proposed in 2017 by Google researchers. It was characterized by better performance than the recurrent neural networks (RNNs) used in machine translation works. The performance was better in terms of training costs as well as translation quality. Similarly, Universal Language Model Fine-tuning (ULMFiT) (Howard & Ruder, 2018) is an effective allocation learning approach, illustrating that pretraining long short-term memory (LSTM) networks using a language modelling goal on a broad and diverse form and then fine-tuning it on a target task was able to produce robust word classifiers with little marked data. Such advances led to the innovation of the popularly used transformers today, namely, BERT (Devlin et al., 2019) and OpenAI’s generative pretrained transformer GPT (Radford & Narasimhan, 2018). A combination of language model pretraining and transformer architecture has enabled transformer models to reduce the requirement for training task-explicit architectures from scratch. It has also overcome the need for benchmarking in NLP by a huge margin. Recently, many other transformer-based language models were released, such as mBERT (Devlin et al., 2019) RoBERTa (Liu et al., 2019) ALBERT (Lan et al., 2019) and DistilBERT (Sanh et al., 2019). These models tried to improve the performance of BERT through slight modifications to the training objective. Recently, the T5 model was presented in Raffel et al. (2020); it is an encoder–decoder model pretrained on a multitask mixture of unsupervised and supervised tasks, for which each task is converted into a text-to-text format. To explore the use of transformer models in abusive content detection studies, we list a sample of the previous studies in Table 5 with the highest F1 score performance for each work.

**Table 5:** Transformer models used for automated abusive content detection and highest F1 performance reported by each work.

Ref.	Language	Transformer Models									F1
		ULMFiT	GPT	BERT	mBERT	ALBERT	DistilBERT	XLM-RoBERTa	ELECTRA	T5	
Nikolov & Radivchev (2019)	English			✓							0.64
Abdellatif & Elgammal (2020)	Arabic	✓									0.77
Rother, Allee & Rettberg (2018)	German	✓									0.80
Arora (2020)	Code-mixed English and Hindi	✓									0.88
Chiu, Collins & Alexander (2022)	English		✓								0.85
Vasantharajan & Thayasivam (2022)	Tamil Code-Mixed	✓			✓		✓	✓			0.74
Fortuna, Soler-Company & Wanner (2021)	English			✓		✓					0.92
Malik, Pang & Van den Hengel (2022)	English			✓					✓		0.97
Sabry et al. (2022)	English									✓	0.83

***BERT*** (Devlin et al., 2019) is a transformer-based machine learning technique for NLP. BERT is a deeply bidirectional, unsupervised language representation that is pretrained using only a plain text corpus. It is also defined as a new language representation model that has been successfully applied to a variety of NLP tasks, obtaining state-of-the-art results for 11 NLP tasks such as sentiment analysis, question answering, and textual entailment. BERT has two models: (1) BERT_BASE_: 12 Encoders with 12 bidirectional self-attention heads, and (2) BERT_LARGE_: 24 Encoders with 24 bidirectional self-attention heads. The rise of using BERT among researchers has been observed to outperform DL in different abusive content detection studies, such as HateBERT (Granitzer, 2020) AraBERT (Djandji et al., 2020) CyberBERT (Paul & Saha, 2020) and HurtBERT (Koufakou et al., 2020).

Moreover, multiple studies claimed that BERT outperformed ML and DL models. A number of studies have investigated BERT’s performance in abusive content detection (Dowlagar & Mamidi, 2020; Alatawi, Alhothali & Moria, 2021), with nearly all authors who compared BERT to other ML and DL models concluding that BERT architecture was superior. Furthermore, BERT achieved the highest F1 score result in different hate speech detection competitions, such as:

**-SemEval-19** Task 6: Liu, Li & Zou (2019) were the top-performing team, achieving an 82.9% F1 score, and they conducted their work with 14k English tweets.

**-SemEval-20** Task 12: Wiedemann, Yimam & Biemann (2020) were the top-performing team by achieving a 92.0% F1 score, and they conducted their work with 14k English tweets.

• Multitask Learning (MTL)

Multitask learning is a learning paradigm that endows the developed models with the human-like abilities of transferring the important learned information between related tasks in what is called inductive transfer of knowledge under the assumption that commonalities exist between the learned tasks. Furthermore, the main advantages of MTL are that it reduces the requirements for large amounts of labelled data, improves the performance of a task with fewer data by leveraging the shared information from the related tasks with more data, and enables the model to be robust to missing observations for some tasks. MTL was used in different studies to detect hate speech and offensive language, such as Djandji et al. (2020) and Abu Farha & Magdy (2020), and according to their findings, the MTL approach achieved the best performance architecture and outperformed all other approaches.

### Evaluation metrics

Most of the studies that were discussed in this article they used assessment criteria to evaluate the obtained result and it is a well-known measurement in ML pipeline. The assessment criteria includes some metrices such as Precision Eq. (3), Recall Eq. (4) ,Accuracy Eq. (5),and,F1-score Eq. (6).The number of accurately categorized positive samples is known as true positive (TP). The number of accurately categorized negative samples is known as true negative (TN). The number of samples misclassified as positive is known as false positive (FP). The number of samples misclassified as negative is known as false negative (FN).

(3) \begin{eqnarray*}& & \text{Precision}= \frac{{T}_{P}}{{T}_{P}+{F}_{P}} \end{eqnarray*}

(4) \begin{eqnarray*}& & \text{Recall}= \frac{{T}_{P}}{{T}_{P}+{T}_{N}} \end{eqnarray*}

(5) \begin{eqnarray*}& & \text{Accuracy}= \frac{{T}_{P}+{T}_{N}}{{T}_{P}+{T}_{N}+{F}_{P}+{F}_{N}} \end{eqnarray*}

(6) \begin{eqnarray*}& & F1-\text{score}=2\mathrm{ \ast } \frac{\text{Precision}\mathrm{ \ast }\text{Recall}}{\text{Precision}+\text{Recall}} \end{eqnarray*}

### Research challenges and opportunities

With increased interest and existing limitations for abusive content automatic detection task. A closer look to the literature, reveals a number of gaps and shortcomings. Therefore, this section will discuss the challenges and limitations exist in the abusive content automatic detection tasks and approaches.

We will highlight important research gaps and suggest future directions in the following sections:

### Fine-grained detection with the quality of the data processing and classification

Most prior studies in this field address the problem as a binary classification task and focus on subtopics of abusive content. Therefore, there is a need to investigate fine-grained categories related to abusive content. Moreover, many researchers recommend extending existing work with a corpus that captures diverse patterns, and there is a need to annotate data to extend the analysis beyond the binary classification problem (Chiril et al., 2022). This type of classification will shed light on many types of abusive content. For instance, a fine-grained hate speech detection on shared task OSACT5 (Mubarak, Al-Khalifa & Al-Thubaity, 2022) used multi types of hate speech and annotate the dataset based on these types.

However, the availability of suitable quality data also remains a challenge. Further investigation includes the discovery of methodologies and techniques that can be used to improve abusive content automatic detection, such as its quality and recent techniques of data collection, preprocessing, and corpus annotation procedures. Another issue is some of the datasets with small data sizes, such as  Albadi, Kurdi & Mishra (2018) and Mulki et al. (2019), which leads to low-resource scenario issues (Şahin, 2022). Thus, it needs some of NLP approaches to improve the performance such as data augmentation and meta learning (Hedderich et al., 2021).

On the other hand, annotation quality and clear guidelines to label the datasets are still challenging problems (Jahan & Oussalah, 2021). Accordingly, designing a standard guideline for the annotator while taking into account evaluation metrics would be helpful, especially in abusive content studies, to avoid bias. In summary, developing new approaches, methods, or algorithms for abusive content dataset creation and annotations is still a challenging task.

### Multimedia content

The majority of abusive content detection problem studies have focused on text analysis. There is a lack of studies that analyze and tackle the multimedia content that spreads hate, such as images, videos, and audios. This absence is due to several challenges, such as technical challenges related to OCR, image recognition, and audio translation  (Vidgen & Derczynski, 2021). Thus, multimedia content opens a new research direction in the form of abusive content detection.

### Multilingual studies

Approximately 51% of all works in this field are performed on English datasets, with a growing fraction of other languages, such as Arabic (13%), Turkish (6%), Greek (4%), and other languages (26%) (Jahan & Oussalah, 2021). However, most existing works tackle the problem in a specific language, and only limited studies have examined multilingual abusive content detection. This is due to the complexity of multilingual studies, which require additional settings to perform some tasks. For instance,  Ousidhoum et al. (2019) used multilingual Babylon embeddings to compute the semantic similarity between words and other technical settings to perform multilingual tasks.

### Dialectal issues in some languages

Multiple words can have the same spelling but have different pronunciations and meanings, creating ambiguity in context. For example, the Arabic language has many dialects, which has led to misunderstandings, especially when we consider the abusive content, since some Arabic terms in a particular region can imply an abusive meaning; however, in another region, such terms are considered common terms (Husain & Ö, 2021).

## Conclusion

This study provides a holistic view of the abusive content automatic detection problem. Firstly, we defined the abusive language and its anti-social behavior categories. Secondly, this article provides a review of the abusive content automatic detection approaches and tasks. In brief, we discussed three research questions to investigate, understand and analyze the existing works in this area. Accordingly, after a comprehensive review we propose a new taxonomy that covers five different aspects and related tasks for the abusive content automatic detection problem. The proposed taxonomy includes, namely, the data and resources, categories and annotation types, pre-processing and feature representation, models and approaches, and the evaluation metrics. Additionally, we investigate the use of different state-of-the-art approaches such as transformer models and its effect in abusive content detection performance. In fact, the power and the rise of pretrained language models such as BERT have gained attention among the research communities. Finally, we discuss the challenges that have been observed among the previous studies and we propose some future directions, with demonstrating the importance of this research area.

## Additional Information and Declarations

### Data Availability

The following information was supplied regarding data availability: No code or raw data is involved in this research as this is a literature review. Generally, all the abusive content dataset that have been discussed in this article are publicly available at GitHub, Kaggle, and Papers with Code. Specific links to search for each dataset are available in Table 2. The transformers models are available at Hugging Face: https://huggingface.co/.
